# Dataset on the abundance of ants and *Cosmopolites sordidus* damage in plantain fields with intercropped plants

**DOI:** 10.1016/j.dib.2016.08.027

**Published:** 2016-08-22

**Authors:** Anicet Gbèblonoudo Dassou, Dominique Carval, Sylvain Dépigny, Gabriel Fansi, Philippe Tixier

**Affiliations:** a CIRAD, Persyst, UPR GECO, TA B-26/PS4, Boulevard de la Lironde, 34398 Montpellier Cedex 5, France; b CARBAP, African Research Centre on Bananas and Plantains, BP 832 Douala, Cameroon; c Laboratory of Biotechnology, Genetic Resources and Plant and Animal Breeding (BIORAVE), Faculty of Sciences and Technology of Dassa, Polytechnic University of Abomey, 01 BP 14 Dassa-Zoumè, Benin; d CIRAD, UPR GECO, F-97285 Le Lamentin, Martinique, France; e Departamento de Agricultura y Agroforesteria, CATIE, 7170, Cartago, Turrialba 30501, Costa Rica

**Keywords:** Associated crops, Ant community, *C. sordidus*, Damages, Plantain, Cameroon

## Abstract

The data presented in this article are related to the research article entitled “Ant abundance and *Cosmopolites sordidus* damage in plantain fields as affected by intercropping” (A.G. Dassou, D. Carval, S. Dépigny, G.H Fansi, P. Tixier, 2015) [1]. This article describes how associated crops maize (*Zea mays*), cocoyam (*Xanthosoma sagittifolium*) and bottle gourd (*Lagenaria siceraria*) intercropped in the plantain fields in Cameroun modify ant community structure and damages of banana weevil *Cosmopolites sordidus*. The field data set is made publicly available to enable critical or extended analyzes.

**Specifications Table**

**Table:** 

**Subject area**	Agronomy, Ecology
**More specific subject area**	Effect of intercropping on banana weevil damages
**Type of data**	Tables, Figures, Text file
**How data was acquired**	Conducting of essay in plantain-based cropping systems in which others crops were planted;
	The weevil damages were observed with Vilardebo method;
	The ant abundances were measured
**Data format**	Raw, Analysed
**Experimental factors**	The three crops maize (*Zea mays*), cocoyam (*Xanthosoma sagittifolium*), and bottle gourd (*Lagenaria siceraria*) were intercropped in a plantain experimental field in order to determine their effects on banana weevil regulation
**Experimental features**	The relationship between the associated plants, predatory ants and banana weevil damages were determined
**Data source location**	Njombé, Cameroon, 4°34′11.33″N; 9°38′48.96″E
**Data accessibility**	The data are available with this article

**Value of the data**

• The data presents the abundance of sampled ants in each crop associated to plantain and could be used by others researchers.
• The weevil damages on the banana bulb were measured by using the Vilardebo method and could be compared to others weevil damage studies.
• This data allows other researchers to extend the statistical analyses.

## 1 Data

**Fig. 1:**
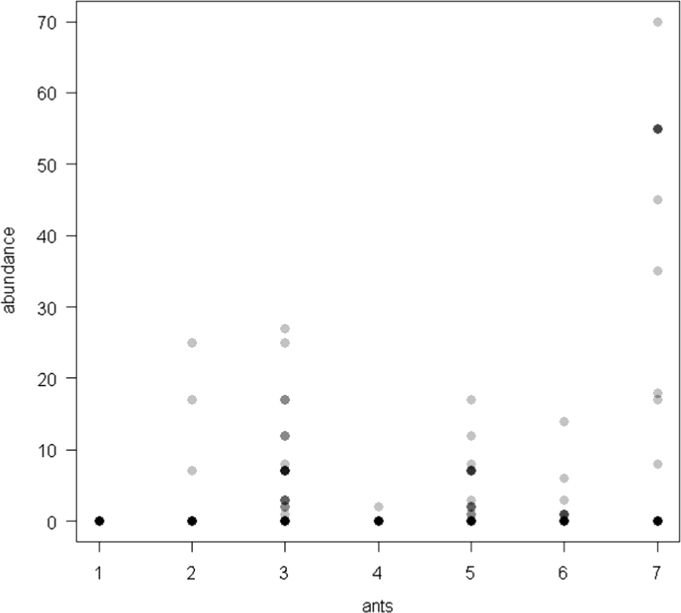
Abundance of different ant taxa in gourd crops associated to plantain, with on the axis 2, 1. *Axinidris* sp., 2. *Camponotus* spp., 3. *Monomorium* spp., 4. *Odontomachus mayi,* 5*. Paratrechina longicornis,* 6. *Pheidole* spp., 7. *Tetramorium* sp.

**Fig. 2:**
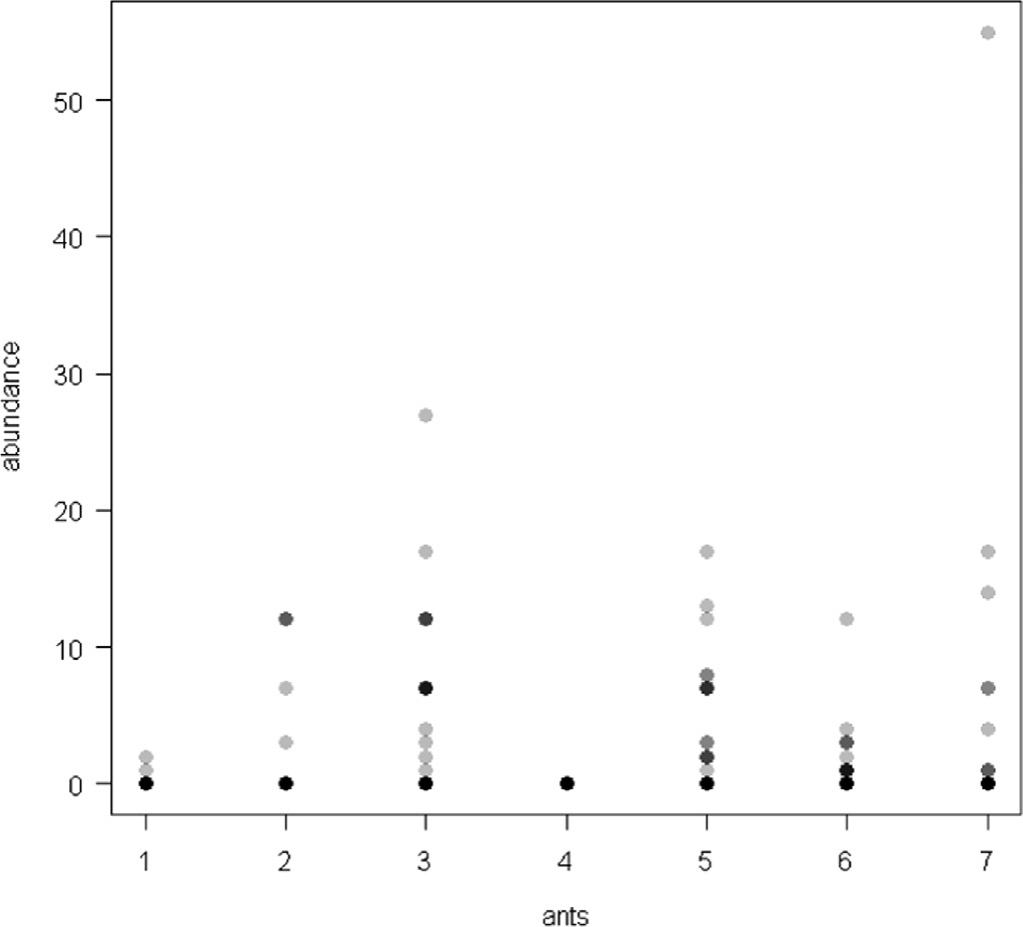
Abundance of different ant taxa in maize crops associated to plantain, with on the axis 2, 1. *Axinidris* sp., 2. *Camponotus* spp., 3. *Monomorium* spp., 4. *Odontomachus mayi,* 5*. Paratrechina longicornis,* 6. *Pheidole* spp., 7. *Tetramorium* sp.

**Fig. 3:**
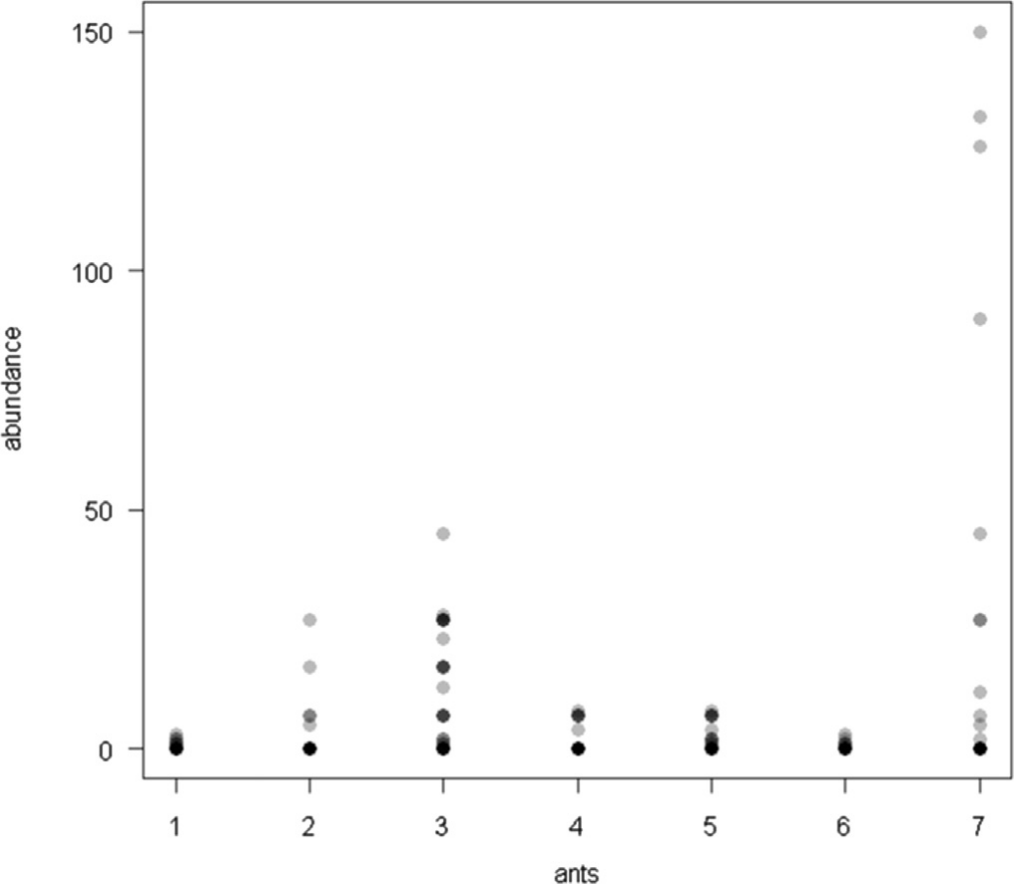
Abundance of different ant taxa in cocoyam crops associated to plantain, with on the axis 2, 1. *Axinidris* sp., 2. *Camponotus* spp., 3. *Monomorium* spp., 4. *Odontomachus mayi,* 5*. Paratrechina longicornis,* 6. *Pheidole* spp., 7. *Tetramorium* sp.

**Fig. 4:**
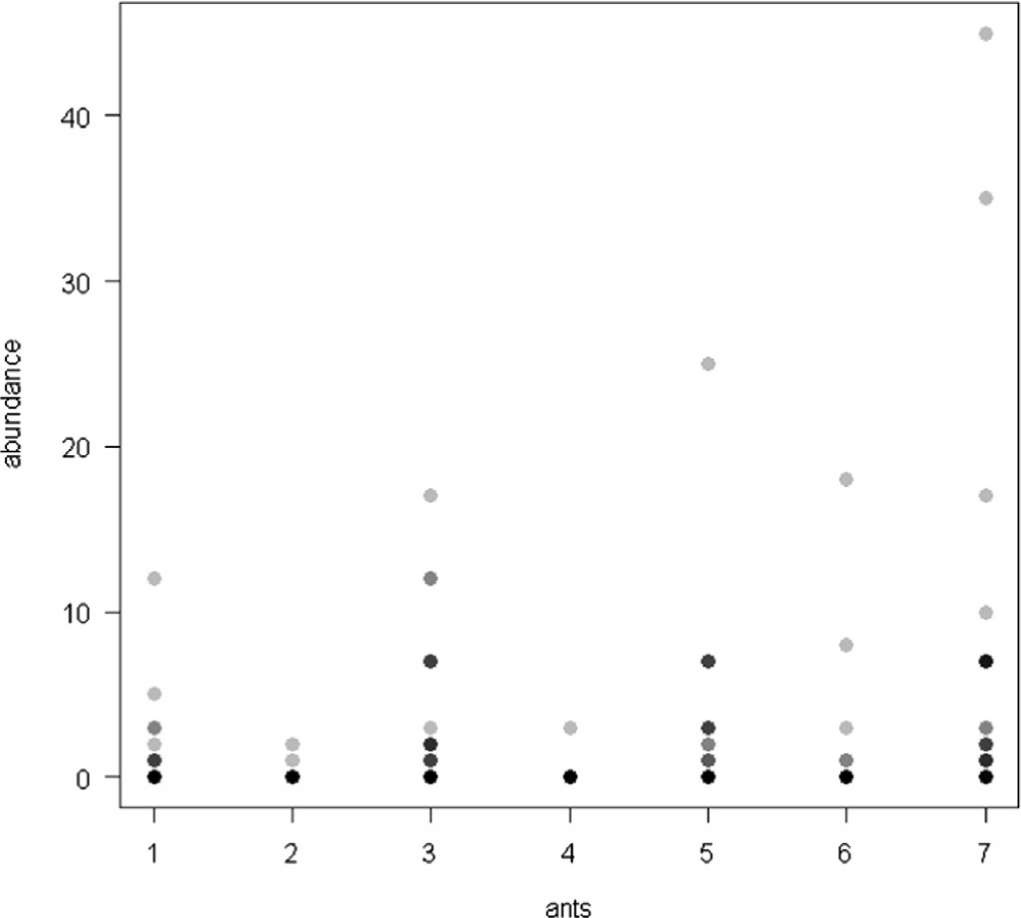
Abundance of different ant taxa in cocoyam-maize crops associated to plantain, with on the axis 2, 1. *Axinidris* sp., 2. *Camponotus* spp., 3. *Monomorium* spp., 4. *Odontomachus mayi,* 5*. Paratrechina longicornis,* 6. *Pheidole* spp., 7. *Tetramorium* sp.

**Fig. 5:**
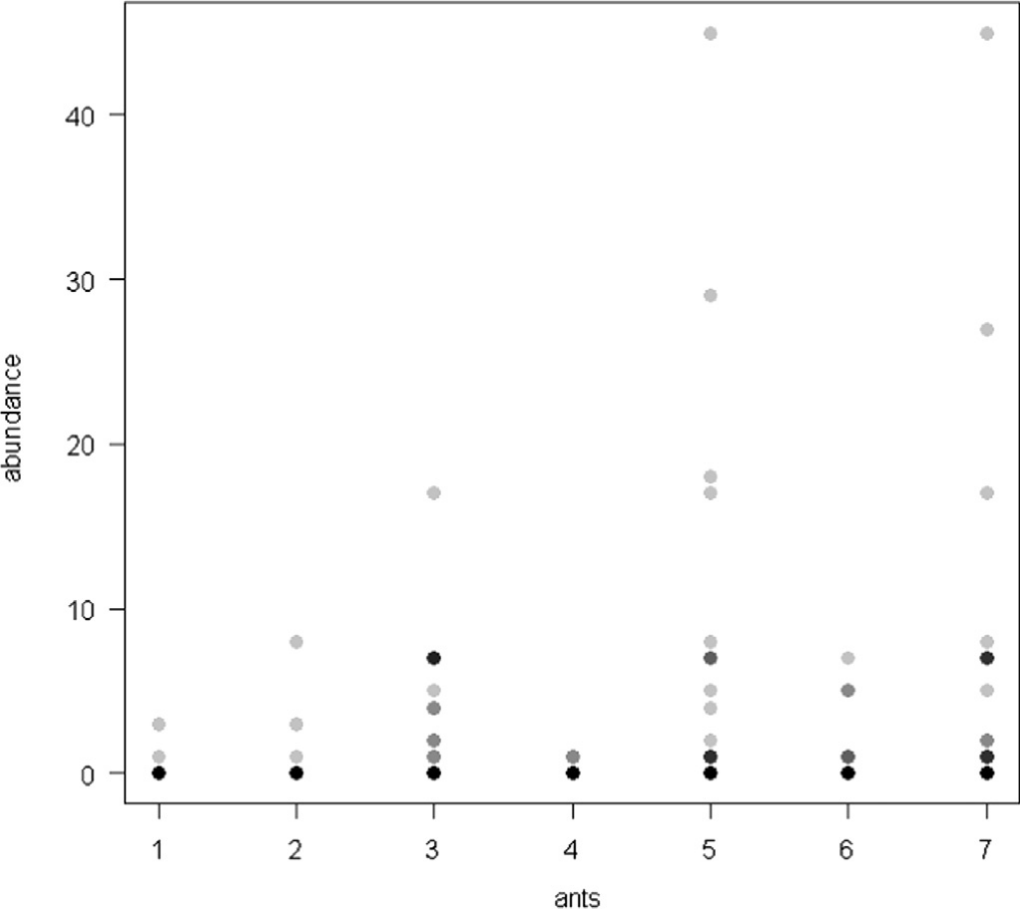
Abundance of different ant taxa in gourd-maize crops associated to plantain, with on the axis 2, 1. *Axinidris* sp., 2. *Camponotus* spp., 3. *Monomorium* spp., 4. *Odontomachus mayi,* 5*. Paratrechina longicornis,* 6. *Pheidole* spp., 7. *Tetramorium* sp.

**Fig. 6:**
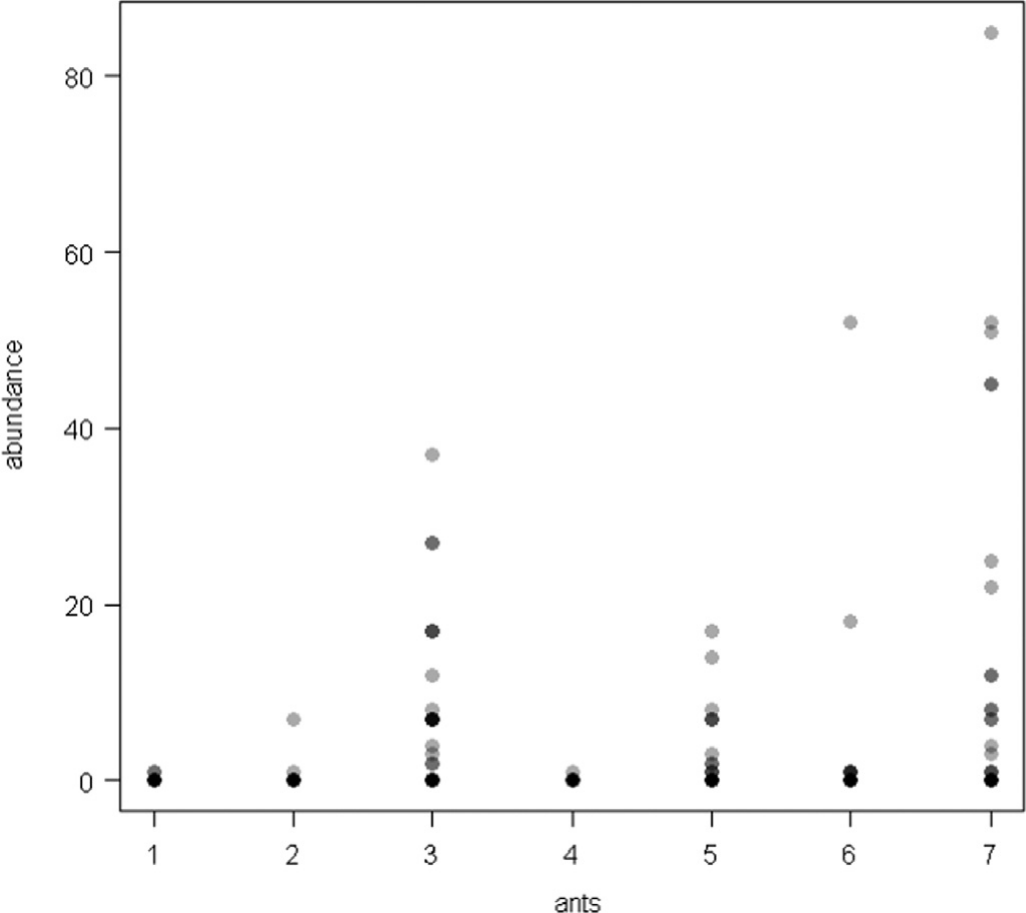
Abundance of different ant taxa in gourd-cocoyam crops associated to plantain, with on the axis 2, 1. *Axinidris* sp., 2. *Camponotus* spp., 3. *Monomorium* spp., 4. *Odontomachus mayi,* 5*. Paratrechina longicornis,* 6. *Pheidole* spp., 7. *Tetramorium* sp.

**Fig. 7:**
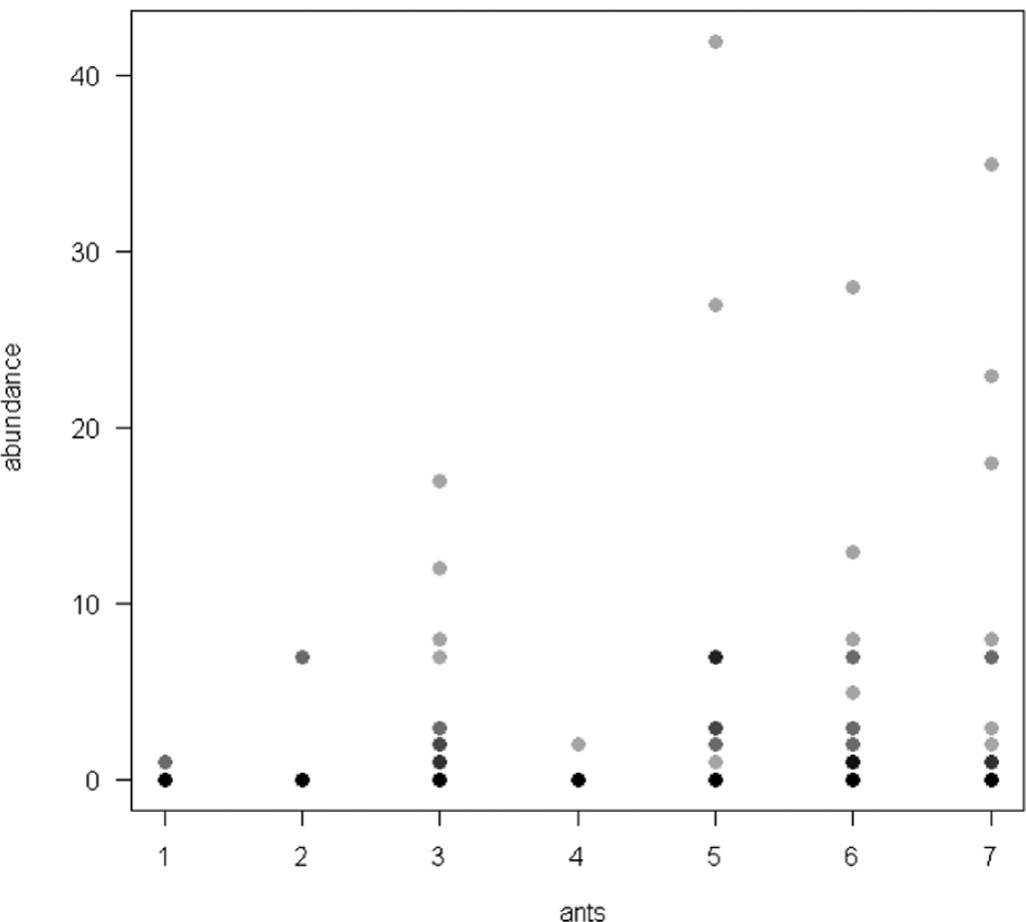
Abundance of different ant taxa in gourd-cocoyam-maize crops associated to plantain, with on the axis 2, 1. *Axinidris* sp., 2. *Camponotus* spp., 3. *Monomorium* spp., 4. *Odontomachus mayi,* 5*. Paratrechina longicornis,* 6. *Pheidole* spp., 7. *Tetramorium* sp.

The dataset of this article provides information on the abundance of ant taxa in the cultivated plants intercropped with the plantain and the weevil damages. The Fig. 1, Fig. 2, Fig. 3, Fig. 4, Fig. 5, Fig. 6, Fig. 7 show the abundance of ant taxa in the crops associated to plantain. Table 1 shows the weevil damage.

## 2 Experimental design, materials and methods

### 2.1 Ant׳s abundance and *Cosmopolites sordidus* damages measurements

The experiments were carried out during two periods: the rainy season and the dry-season in Cameroon. Three crops (bottle gourd, maize and cocoyam) and theirs combinations were intercropped with the plantain. In each experimental unit, to measure the abundance of ants, we used the attractive traps placed at 0.5 m of each plantain plant and alternated the side at each plantain plant [1]. These attractive traps were composed of 30×30 cm white ceramic plates, each of which had at its centre a 4-cm spot of bait composed of honey mixed with canned tuna [1], [2]. The bait trap, which was designed to detect the abundance of ants, was deployed for 30 minutes before ants were collected with an aspirator. Ants were counted in digital photographs of ceramic plates. At the end of essay, we evaluated the damages of larvae on the banana bulb with Vilardebo [3] method.

## Funding sources

This work is part of a Ph.D. thesis of Anicet Dassou and was funded by CIRAD, France (AIRD grant) and the C2D project.

## Figures and Tables

**Table 1:** Measure of *Cosmopolites sordidus* damages on the plantain by treatment according to the Vilardebo method.

**Fields**	**Gourd**	**Maize**	**Cocoyam**	**Cocoyam maize**	**Gourd maize**	**Cocoyam gourd**	**Cocoyam gourd maize**
1	60	60	0	20	20	60	40
1	20	100	40	60	30	10	0
1	60	20	100	60	60	100	40
1	60	40	10	0	60	60	40
1	10	10	10	60	60	60	0
1	60	60	30	20	60	60	10
1	20	10	20	10	60	30	0
1	100	40	30	20	60	100	30
2	60	60	30	20	30	40	60
2	0	60	30	10	20	60	5
2	30	30	10	40	20	60	20
2	0	40	40	40	20	100	20
2	60	40	20	40	30	10	40
2	20	20	30	0	10	60	30
2	40	40	5	20	10	100	20
2	60	40	0	40	10	60	20
3	20	10	5	0	30	30	0
3	20	100	20	20	60	20	10
3	40	100	10	40	30	40	0
3	30	40	100	10	30	20	10
3	60	100	60	100	30	60	30
3	30	60	60	0	60	100	5
3	10	40	60	60	60	20	60
3	20	40	40	20	60	40	20
4	20	30	20	20	20	20	20
4	5	0	0	10	5	5	30
4	10	5	20	20	20	30	30
4	20	10	20	30	20	10	20
4	40	100	20	60	60	10	100
4	20	30	60	20	100	20	60
4	40	0	0	60	60	30	60
4	60	30	30	20	20	40	60

## References

[1] Dassou A.G., Carval D., Dépigny S., Fansi G.H., Tixier P. (2015). Ant abundance and *Cosmopolites sordidus* damage in plantain fields as affected by intercropping. Biol. Control.

[2] Dassou A.G., Dépigny S., Canard E., Vinatier F., Carval D., Tixier P. (2015). Contrasting effects of plant diversity across arthropod trophic groups in plantain-based agroecosystems. Basic Appl. Ecol..

[3] Vilardebo A. (1973). Le coefficient d׳infestation, critere d׳evaluation du degre d׳attaques des bananeraies par *Cosmopolites sordidus* Germar. le charancon du bananier. Fruits.

